# Language in Context: MEG Evidence for Modality-General and -Specific Responses to Reference Resolution

**DOI:** 10.1523/ENEURO.0145-16.2016

**Published:** 2016-12-30

**Authors:** Christian Brodbeck, Laura Gwilliams, Liina Pylkkänen

**Affiliations:** 1 Department of Psychology, New York University, New York, NY 10003; 2 Department of Linguistics, New York University, New York, NY 10003; 3 NYU Abu Dhabi Institute, New York University Abu Dhabi, Abu Dhabi, United Arab Emirates

**Keywords:** language comprehension, MEG, reference, situation model, visual world

## Abstract

Successful language comprehension critically depends on our ability to link linguistic expressions to the entities they refer to. Without reference resolution, newly encountered language cannot be related to previously acquired knowledge. The human experience includes many different types of referents, some visual, some auditory, some very abstract. Does the neural basis of reference resolution depend on the nature of the referents, or do our brains use a modality-general mechanism for linking meanings to referents? Here we report evidence for both. Using magnetoencephalography (MEG), we varied both the modality of referents, which consisted either of visual or auditory objects, and the point at which reference resolution was possible within sentences. Source-localized MEG responses revealed brain activity associated with reference resolution that was independent of the modality of the referents, localized to the medial parietal lobe and starting ∼415 ms after the onset of reference resolving words. A modality-specific response to reference resolution in auditory domains was also found, in the vicinity of auditory cortex. Our results suggest that referential language processing cannot be reduced to processing in classical language regions and representations of the referential domain in modality-specific neural systems. Instead, our results suggest that reference resolution engages medial parietal cortex, which supports a mechanism for referential processing regardless of the content modality.

## Significance Statement

Reference resolution is an elementary mechanism for language comprehension, connecting language meaning to pre-existing knowledge. It is unknown whether reference resolution depends on brain mechanisms specific to the modality of the referents (for example, whether they are visual or auditory objects) or whether our brains use a modality-general mechanism for linking meanings to referents. Here we show using source-localized magnetoencephalography that reference resolution is associated with a response in the medial parietal lobe, independent of referent modality, supporting a modality-general mechanism for reference resolution. An additional response associated with resolving reference to auditory objects in auditory cortex suggests that modality-specific representations of the referents are also involved.

## Introduction

A crucial precondition for understanding a sentence in context is identifying the entities that the sentence is about. This was demonstrated in a classic study showing how a text passage that appears incomprehensible when presented in isolation becomes perfectly natural when presented after a picture that provides meaningful referents for the text (Bransford and Johnson, 1972). However, much of the research on the neural basis of language comprehension ignores this referential dimension, studying generic sentences presented without a specific context. Here we report a study in which we deliberately manipulated the relationship between background knowledge and linguistic expressions to uncover the neural basis of successful reference resolution.

Unlike most laboratory experiments, language comprehension in the real world takes place in a rich context. Language comprehenders must not only decode the literal meaning of a message, but also connect it to mental models representing what the message is about (Graesser et al., 1997). A body of evidence suggests that such models are connected to modality-specific cognitive systems. For example, even when participants are looking at a blank screen while listening to stories, their eye movement patterns reflect spatial configurations described in the language input (Spivey and Geng, 2001; Altmann, 2004; Altmann and Kamide, 2009). Furthermore, electroencephalography (EEG) data suggest that readers resolving reference to an item on a previously seen visual display access a retinotopic representation (Brodbeck et al., 2015).

**Fig. 1.**
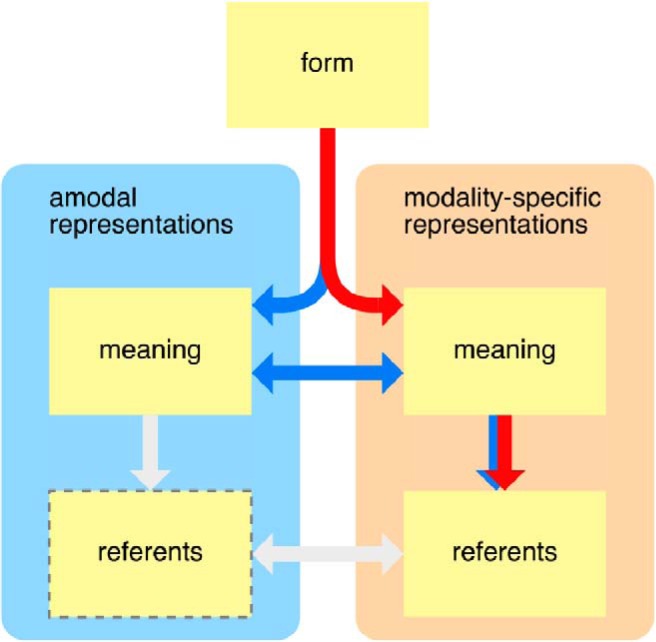
Modality-specific and modality-general representations. Possible flow of information between modality-general and modality-specific representations of both linguistic meaning and referents. Information originates at the top in linguistic form information, i.e., written or spoken words. This input triggers retrieval of meanings, which in turn are used to find referents. Some theories suggest the existence of a “semantic hub” that is involved in processing semantic representations regardless of the modality of their content (Patterson et al., 2007), and others suggest that meaning is exclusively represented in modality-specific brain systems (Pulvermuller, 2013). Both of these theories are potentially compatible with a model in which referents are represented exclusively in modality-specific brain systems (blue and red arrows, respectively). This hypothesis would suggest that the “referents” box at the bottom left can be removed from the diagram, since referential processing is constrained to modality-specific representations.

These observations resonate with theories of embodied meaning, according to which meaning is represented in the same cognitive systems that also process sensory information (Barsalou, 1999; Hauk et al., 2008). Theories of strong embodiment go as far as proposing that lexical meanings are represented in sensory regions (Pulvermuller, 2013), implying that the task of mapping meanings to referents could be performed by modality-specific systems exclusively (red path in Fig. 1). But even if lexical semantics involves amodal regions, it is still possible that situation models, in which specific referents are represented, are constrained to sensory-specific cortices (blue path in Fig. 1). In such a model, amodal regions should be sensitive to lexical, but not referential, properties of language input.

Alternatively, reference resolution could involve an amodal or modality-general mechanism that mediates between lexical and referential meanings. Such a mechanism could be related to amodal discourse representations (Graesser et al., 1997), but it might also be required by embodied theories of meaning—for example, to coordinate referents of different modalities.

A number of functional MRI (fMRI) and positron emission tomography (PET) studies have investigated linguistic contrasts that involved referential properties. Coherent language, which involves repeated reference to the same entities, consistently activates medial frontal and medial parietal regions in addition to classic perisylvian language areas (Ferstl et al., 2008). More specifically, medial as well as lateral parietal areas were more active in response to sentence pairs introducing a conjoined subject (e.g., *Jeremy and Roger*) compared with individually introduced referents, suggesting that these regions might be involved in creating and tracking discourse referents (Boiteau et al., 2014). Medial and lateral parietal, as well as frontal, areas were also activated by sentences containing unresolved referential ambiguities (Nieuwland et al., 2007). Although these results point to a number of brain regions that could be involved in referential processing, the low temporal resolution of fMRI and PET makes it difficult to link brain activation to specific linguistic processes, which follow each incoming word in a rapidly cascading fashion. Finally, none of these studies explicitly manipulated the modality of the referents, so their results could be related to imagery associated with language comprehension.

**Fig. 2.**
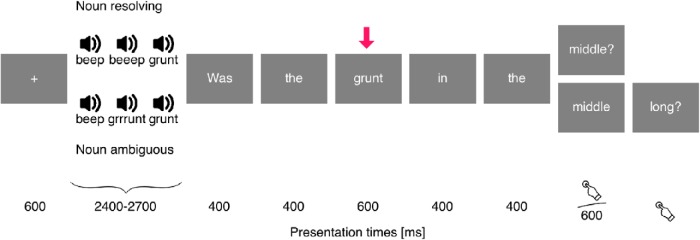
Design for auditory referential domains. Time proceeds from left to right, with vertical offset indicating elements that differ between conditions. The arrow indicates the target word for analysis. The upper sequence illustrates a trial in which the target word *grunt* resolves reference, whereas the lower sequence illustrates a trial in which it does not, and reference is resolved by adding the prepositional phrase *in the middle*. Below the displays, presentation time of each frame is indicated in milliseconds.

**Fig. 3.**
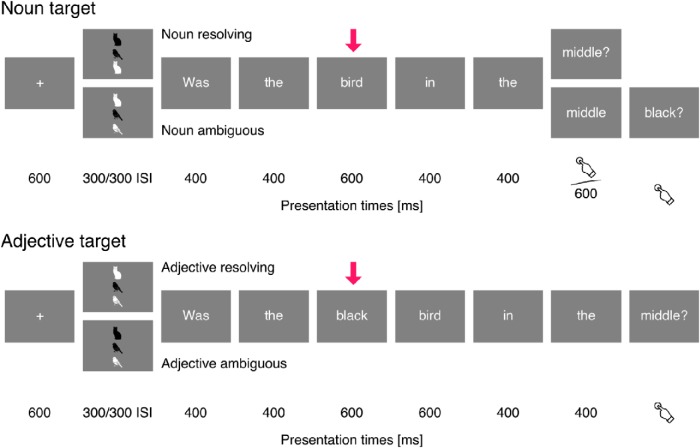
Design for visual referential domains. Time proceeds from left to right, with vertical offset indicating elements that differ between conditions. The arrow indicates the target word for analysis. For both trials with noun targets and trials with adjective targets, the upper sequence illustrates a trial in which the target resolves reference, and the lower sequence illustrates a trial in which reference is resolved later in the sentence. Below the displays, presentation time of each frame is indicated in milliseconds.

Reference resolution itself is inherently difficult to separate from other cognitive processes associated with managing referents, such as retrieving referents from memory and locating them in the environment (see Discussion). Here we tried to create an experimental situation that minimizes memory retrieval and scanning of the environment by presenting simple referential contexts immediately preceding linguistic stimuli that contained referential expressions. We used magnetoencephalography (MEG) to directly contrast reference resolving words with nonresolving controls. To locate a response involved in reference resolution independent of the modality of the referential domain, we constructed parallel conditions with auditory and visual referential domains (see Figs. 2 and 3). We predicted two possible response patterns: regions that track the set of possible referents should exhibit more activation for the ambiguous condition, in which the expression is compatible with two referents; and regions that become active when reference is resolved should exhibit more activity in the resolving condition.

## Materials and methods

### Participants

We collected data from 26 right-handed native English speakers recruited from the community on the New York University–Abu Dhabi campus. Two participants were excluded because of excessive artifacts leading to <70% good trials, leaving 24 participants for the final analysis (18 female, mean age 24 years, range 19–50). New York University–Abu Dhabi, is an English-speaking university located just outside the city of Abu Dhabi. Our participants were thus immersed in an English-speaking environment that involves little contact with local languages unless students and staff actively seek it out. Of the 24 participants in the final analysis, 16 had grown up speaking only English, whereas 8 had grown up bilingual. The second native languages were Hindi, Japanese (2), Malayalam, Mandarin, Spanish, Urdu, and Vietnamese. The protocol was approved by the Institutional Review Board of New York University–Abu Dhabi, and all participants provided written consent before beginning the experiment.

### Materials

Each trial consisted of presentation of a referential domain, followed by a question about the domain, presented word for word (see Figs. 2 and 3). Participants’ task was to answer questions such as *Was the grunt in the end?* by pressing one of two response buttons. Target stimuli were referential expressions that were identical in their linguistic surface properties but differed in whether they resolved the reference, for example, *the grunt* in a context that contained one or two grunts.

### Auditory referential domains

For auditory referential domains, we selected 10 sounds with monosyllabic names that were easily identifiable and for which short (∼100- to 300-ms) and long (∼500- to 600-ms) variants were clearly distinguishable. Nine sounds (bark, buzz, caw, chirp, cluck, croak, honk, mew, and splash) were extracted from sounds available under a creative commons license (http://freesound.org), and one (beep) was created from a 1000-Hz sine wave.

Fig. 2 shows a sample trial for the auditory referential domains. Each referential domain consisted of three sounds, played sequentially. All domains were constructed such that two sounds shared the name and two other sounds had the same length. Presentation of the domain started with a fixation cross for 600 ms, followed by the three sounds. For long sounds, the stimulus-onset asynchrony was 1000 ms; for short sounds, it was 700 ms. The domain was followed by the question in serial visual presentation, with function words presented for 400 ms and content words presented for 600 ms. The last word of the question was ended with a question mark and stayed on the screen until the participant made a yes/no response via button press.

The target for analysis was the first noun in the question, marked with an arrow in Fig. 2. In trials of the reference-resolving condition, the noun named a unique sound, i.e., the sound that did not share its name with another sound in the domain. In control trials, the noun did not resolve reference because it was compatible with two sounds in the domain that shared the same name. In this case, the noun was followed by a prepositional phrase that used the referent’s location for disambiguation, for example *the grunt in the middle*. When the noun resolved reference, the question continued asking for the temporal location of the sound, for example *Was the grunt in the middle?* In control trials, the question asked for the length of the sound, as in *Was the grunt in the middle long?* The correct answer to the question was “yes” on exactly half of the trials, counterbalanced between conditions. This design assured that conditions were indistinguishable up to the critical noun.

When hearing one of the sounds by itself, it might have been difficult to judge its length, i.e., whether it was the long or the short token. However, because the question asked only for the length of the sound in the control condition, this question always followed domains that included both the long and the short version of the same sound, allowing for a direct comparison of the two versions.

A list of all possible referents was generated by permuting referent name (10 nouns), referent length (long or short), and referent location (first, second, or third sound). One such list was assigned to the resolving noun condition, another to the ambiguous noun condition, and a third was split between the two conditions to produce 30 trials in each cell of the reference × location design. The remaining elements of each trial were filled in using a balanced randomization procedure. The same trials were presented to each participant, but the order was randomized for each session.

### Visual referential domains

For the visual referential domains, we selected 10 objects with monosyllabic names that could be used to generate easily identifiable outline images (truck, star, house, car, fish, pig, tree, bird, boat, cat). Outlines were created based on photos using the Inkscape vector drawing application. To match visual referential domains in complexity to the auditory domains, where sounds were either long or short, we chose just two colors, black and white.

Trials were constructed analogously to the trials with auditory referential domains. Fig. 3 shows a sample trial for each condition. Each referential domain consisted of three objects arranged vertically. All domains were constructed such that two objects shared the shape, and two other objects shared the color. Each trial started with presentation of a fixation cross for 600 ms, followed by the referential domain presented for 300 ms, and the question after an interstimulus interval of 300 ms. Presentation of the questions followed the same protocol as for auditory referential domains.

In visual domain trials, the form of the referential expression was varied as an additional factor. In half of the trials, referential expressions were constructed in parallel to the auditory domain condition, with resolving noun contrasted with ambiguous nouns followed by a prepositional phrase specifying the location (e.g., *the bird* versus *the bird in the middle*). In the other half of the trials, the referential expression was an adjective-noun phrase, such as *the black bird*. In those expressions, the adjective was the target. In a domain with only one black item, the adjective resolved reference, whereas in trials with two black items, the noun did. Because the location was never included in the referential expression, these questions always asked for the location of the referent. Referential expressions with adjective targets were not included with auditory referential domains because sounds would have been harder to distinguish based on length alone.

Including reference-resolving adjectives gave us an additional distinction between reference resolution at complete and incomplete linguistic phrases. There is evidence that the status of an expression as a complete linguistic phrase interacts with referential processing. Eye tracking studies suggest that listeners predict whether a potentially complete noun phrase will be followed by a prepositional phrase or not, based on whether they could resolve reference; i.e., if a noun phrase is compatible with several potential referents, they expect additional information to disambiguate between competing referents (Tanenhaus et al., 1995; Spivey et al., 2002). A response to reference-resolving nouns (*the bird*) could thus also indicate the completion of a linguistic phrase, since the comprehender would expect the phrase to be elaborated in the control condition (*the bird in the middle*). Adjectives did not entail this contrast, since they were always followed by a noun (*the black bird*). In contrast, referential processing should happen at incomplete phrases too. Eye tracking as well as EEG evidence suggest that language comprehenders use the information in reference-resolving adjectives. For example, when participants are instructed, *Touch the starred yellow square* in a context with only one starred item, they look at the target item shortly after the word *starred* (Eberhard et al., 1995; Sedivy et al., 1999). Similarly, readers presented with a referential expression in which the adjectives allows resolving reference to an object on the left or the right side of a visual display exhibit an event-related potential that is sensitive to the location of the referent starting ∼333 ms after adjective onset (Brodbeck et al., 2015). Including adjectives as targets thus allowed us to distinguish between phrasal and referential processing.

The same procedure as for auditory domains was used to create 30 trials per condition in the reference (target-resolving or ambiguous) by location (top, middle, bottom) by target (noun, adjective) design (Fig. 3).

### Lexical variables

The present study was specifically designed to assess reference resolution in different contexts: in auditory and visual referential domains, and with information conveyed by a noun or an adjective. Stimuli were developed with a focus on creating natural referential situations. Within each context, target words were identical between the resolving and ambiguous target conditions, and since our hypotheses did not pertain to main effects of context, matching target words between different contexts was not a priority. Between contexts, target items differed both in variability (two adjective tokens were used, whereas 10 noun tokens were used per modality) and in word frequency. Lexical frequency was assessed using the contextual diversity variable in the SUBTL corpus (Brysbaert and New, 2009), a simple count variable that reflects in how many of 8388 films and television episodes this word occurred at least once. Nouns describing auditory objects were less frequent than nouns describing visual objects (range 6–383 for auditory nouns and 1056–6040 for visual nouns); visual adjectives fell in the range of the visual nouns (*white*, 3355; *black*, 3190).

### Procedure

Before the recording session, participants’ head shape was digitized using an optical FastSCAN scanner (Polhemus). The scan included the positions of five marker coils that were later attached to the participant’s head. At the beginning and end of each experimental session, the position of those marker coils was recorded relative to the MEG sensors, and this record was later used to coregister the head shape relative to the MEG sensor positions for the source localization procedure.

Participants were familiarized with the task and introduced to the MEG recording procedure. MEG acquisition took place in a magnetically shielded chamber, in which participants were lying in a supine position with their head resting in the helmet-shaped dewar containing the SQUIDs. Stimuli were presented with PsychoPy (RRID:SCR_006571), projected onto a screen above the participant’s head. Participants were allowed to complete as many practice trials as they needed to feel comfortable with the task with both visual and auditory referential domains. They were asked to move as little as possible and to try not to blink while reading the questions. They were given the option to pause the experiment after every trial by pressing the response button twice when giving their answer.

Stimuli were presented in blocks of 45 trials. Auditory and visual referential domains were presented in separate interleaved blocks, and the beginning of each block announced the modality of the domains, so that participants knew which modality to attend to. Within auditory and visual domain trials, the order of trials was randomized. Because there were twice as many visual as auditory trials, blocks were presented in V-A-V-V-A-V-V-… sequence. After each block, participants had the opportunity to take a brief self-terminated break.

For three participants, the MEG session was interrupted. One session was interrupted by a fire alarm, one because of excessive tiredness, and one for a bathroom break. All affected participants finished the experiment, on the same day (two participants) or a different day (one participant). Marker positions from the first session were used online to match the head position in the second session closely to the first session.

### Data acquisition and analysis

Continuous MEG was recorded with a 208-channel axial gradiometer system (Kanazawa Institute of Technology) at a sampling rate of 1000 Hz. Data were bandpass filtered between 0.1 and 200 Hz online.

### Preprocessing

Nonperiodic environment noise was removed from the raw data by regressing the signal against 16 orthogonal reference sensors using the continuously adjusted least-squares method (Adachi et al., 2001). Data were then converted to the FIFF format and processed with mne-python (v. 0.11, RRID:SCR_005972; Gramfort et al., 2013, 2014) and additional tools available in Eelbrain (v. 0.22.1, RRID:SCR_014661)

Bad channels were excluded from analysis based on visual inspection. Data were low-pass filtered at 40 Hz. Epochs from –100 to 600 ms relative to onset of the target words were extracted and screened for artifacts. Epochs exceeding a ±2000 femto-tesla absolute threshold were removed automatically. In addition, subthreshold epochs were manually removed if channels close to the eyes significantly diverged for longer than 300 ms, indicating presence of an ocular artifact. If an individual channel within an epoch significantly deviated from the group average, it was interpolated just for that epoch. Good epochs were downsampled to 200 Hz, averaged per condition, and baseline corrected with the 100-ms prestimulus interval.

### Source estimation

Because structural MRI datasets were not available for our participants, we used the “fsaverage” average brain model included with FreeSurfer (RRID:SCR_001847) for source localization. To provide better localization accuracy, the fsaverage model was scaled to match each individual’s head shape, acquired before the experiment (see Procedure). The complete coregistration procedure proceeded as follows. First, the fsaverage head model was aligned with the participant’s head shape by matching the nasion position. The fsaverage head model was then modified using rotation and uniform scaling, with the nasion as center, to minimize the distance of the pre-auricular points on the two head models using an iterative least-squares procedure. Finally, minor adjustments to the translation were made as necessary to fit the head scan to the fsaverage head shape while taking skull-external properties (e.g., amount of hair) into consideration.

The source space was defined on the white matter surface with the topology of a recursively subdivided icosahedron (“ico-4” option). Sources lying in the corpus callosum and subcortical structures were excluded based on the PALS-B12 atlas (Van Essen, 2005). Anatomical areas were labeled based on the Desikan–Killiany atlas (Desikan et al., 2006). Pre- and postrolandic gyri and the insula, where we did not expect any meaningful effects, were excluded from the analysis, resulting in approximately 1950 source locations in each hemisphere.

For each subject, a separate inverse solution was computed based on the covariance matrix from the 100-ms baseline period (good trials only). Brain activity was estimated across space and time using distributed noise-normalized minimum norm current source estimates (Dale et al., 2000). Noise-normalization provides advantageous localization accuracy over raw minimum norm estimates for nonsuperficial sources (Hauk et al., 2011), which was relevant because of the midline regions that were of interest (see Introduction). For each source location, current was estimated at three orthogonal dipoles to form a 3D current vector, of which only the length was retained to provide a nondirectional measure of activation. These orientation-free source estimates take into account that the fsaverage brain, while providing approximate locations of anatomical features, might not accurately reflect precise individual cortical folding patterns.

Because source activity was estimated on the same brain model, though scaled to different sizes, estimates for different subjects were directly comparable without morphing data from one brain model to another.

### Statistical analysis

The primary statistical analysis was based on a mass-univariate analysis with spatiotemporal cluster-based permutation tests (Holmes et al., 1996; Maris and Oostenveld, 2007). Source estimates of condition averages for each subject entered the analysis. A repeated measures ANOVA *F*-value was computed for each source at each time point in a prespecified anatomical area and time window. This *F*-map was thresholded at an *F*-value corresponding to an uncorrected *p*-value of 0.05. Clusters were formed based on direct adjacency in space and time. For each cluster, the exceedance mass was calculated (the sum of all *F*-values in the cluster). The same procedure was repeated in 10,000 random permutations of the original data, shuffling condition labels within subject to take into account the within-subject nature of the design. For each permutation, the largest cluster mass value was retained to form a nonparametric estimate of the distribution of the largest cluster mass value under the null hypothesis that condition labels are exchangeable. Finally, a *p*-value was computed for each cluster in the original *F*-map as the proportion of permutations that yielded a cluster with a larger mass than the cluster under question. We report all clusters that reached a *p*-value of 0.05 or smaller.

To describe the pattern of activation in each significant cluster, we then extracted and plotted the time course of activation, as well as the average activation in the cluster. For the time course, we created a region of interest (ROI) encompassing all sources that were part of the cluster at any point in time. For the average activation, we used the cluster as a spatiotemporal mask to extract a single average activation value for each subject and condition. All plots indicate within-subject standard errors as a measure of variability (Loftus and Masson, 1994).

### Analysis design and parameters

The goal of the primary analysis was to test whether reference resolution was associated with a certain brain response across different contexts, or whether there was a response to reference resolution that depended on the context, i.e., the modality of the referential domain or the target word type. We thus performed an initial 2 × 3 × 3 ANOVA with factors reference (reference resolving versus ambiguous target), condition (auditory domain with noun targets, visual domain with noun targets, visual domain with adjective targets), and location of the referent in the domain (first/top, middle, last/bottom).

**Fig. 4.**
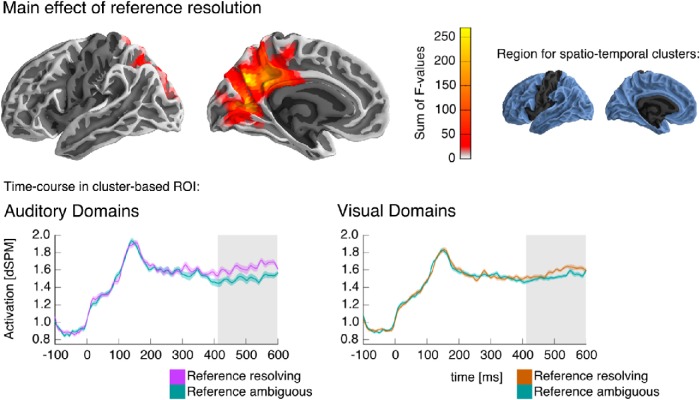
Full-model ANOVA results. The ANOVA analysis revealed a significant spatiotemporal cluster for the main effect of reference resolution in the medial parietal lobe of the left hemisphere, indicating an activity increase associated with reference resolution starting at 415 ms after target word onset. ***Top left***, Anatomical extent of the cluster, shown on a lateral and a medial view of the left hemisphere. Each source that was part of the cluster at any time point is color coded, indicating the sum of *F*-values over time, i.e., how much that particular source contributed to the cluster’s mass. ***Top right***, The anatomical region included in the test is indicated in blue; the left hemisphere is shown for illustration, but the test also included the corresponding area of the right hemisphere. ***Bottom***, The time course of activation in the ROI defined by the cluster in response to the target words, shown separately for auditory and visual referential domains for illustration purposes. Time 0 ms corresponds to the onset of the visual presentation of the target word. The time course plots indicate within-subject standard errors. The time region covered by the cluster is indicated with gray shading (415–600 ms).

Prior fMRI work on referential language processing (Almor et al., 2007; Nieuwland et al., 2007; Boiteau et al., 2014) and coherent language (Ferstl et al., 2008) suggests a large number of brain regions with possible involvement in reference resolution. To account for this, our initial analysis included the cortex of both hemispheres except for the pre- and postrolandic gyri and insula as defined in the Desikan–Killiany atlas (Desikan et al., 2006). This region is illustrated at the top right of Fig. 4.

We used a time window of 200–600 ms relative to target word onset for this analysis. Traditional models from electrophysiology have suggested that lexical access occurs shortly after 300 ms relative to a written word presentation (Pylkkänen and Marantz, 2003; Grainger and Holcomb, 2009). Because a word has to be recognized before it can be related to the referential domain, this estimate would provide a lower bound for expected effects of referential processes. However, more recent evidence suggests that access to lexical and semantic information can happen by ∼200 ms (Hauk et al., 2012), and shortly after that effects of semantic composition can be detected (Bemis and Pylkkänen, 2011). We thus used a large time window to include potential early effects.

Based on the result of a main effect of reference resolution in the initial analysis, we then performed a conjunction analysis to confirm the presence of a response regardless of the modality of the referential domain (Nichols et al., 2005). First, two *t*-maps were created, one comparing reference resolving to ambiguous targets in auditory domains and the other in visual domains. The conjunction map was defined as the element-wise minimum of those two maps (*t*_conjunction_ = min(*t*_reference auditory_, *t*_reference visual_). Clusters were formed using a threshold equivalent to the one-tailed *p*-value of 0.05. The significance of clusters was assessed with a permutation test, as described above, by repeating the analysis in 10,000 permutations with shuffled condition labels.

After the result of a main effect of reference resolution in the medial posterior region of the left hemisphere, we performed a *post hoc* test in a more constrained region to ascertain the absence of an interaction effect that would qualify the result for the main effect. For this purpose, we defined a spatial region, slightly larger than the main effect cluster, using labels from the Desikan–Killiany atlas (Desikan et al., 2006). The labels included were the paracentral lobule, precuneus, cuneus, pericalcarine cortex, lingual gyrus, and posterior and isthmus divisions of the cingulate gyrus. These roughly correspond to Brodmann areas 23, 26, 29–31, the medial aspects of 1–5 and 7, and the medial superior parts of 18 and 19.

Finally, we also assessed modality-specific effects of reference resolution for auditory and visual domains in more targeted tests. Targeted anatomical search regions were defined based on labels in the Desikan–Killiany atlas (Desikan et al., 2006). For auditory domains, we defined a search region broadly construed as auditory cortex. This region included the transverse temporal, superior temporal and supramarginal gyri of both hemispheres (roughly, Brodmann areas 22 and 40–42). Several lines of research suggest that auditory objects are represented in the superior temporal gyrus adjacent to primary auditory areas (Ding and Simon, 2012; Bizley and Cohen, 2013; Giordano et al., 2013). We included supramarginal gyrus because activation associated with (nonlinguistic) auditory imagery tends to extend into this region (McNorgan, 2012). We also performed an analogous analysis for visual referential domains in the occipital lobe as defined by the PALS-B12 atlas (Brodmann areas 17–19), although this last test was more tentative, because brain activity related to concurrent visual presentation of the sentence stimuli could interfere with our ability to record activity associated with visual referents.

With one exception, we are not reporting any analyses of brain responses to events subsequent to the targets, because our stimuli were not designed to analyze these. As illustrated in Figs. 2 and 3, after the target word there were systematic differences between stimuli with reference-resolving targets and stimuli with ambiguous targets. The possible exception is nouns in the visual, adjective-noun phrase condition (see Fig. 3, bottom). Here, nouns following ambiguous adjectives resolve reference, whereas nouns following reference-resolving adjectives are in a referential sense redundant. However, this prediction is somewhat weakened because EEG data suggest the possibility of a reference resolution–like response to nouns in adjective-noun phrases even after adjectives resolve reference (Brodbeck et al., 2015). This could, for example, reflect a process of double-checking that the noun fits with the referent. We analyzed the response to these nouns analogously to the other analyses described above, except that we used –100- to 1200-ms epochs relative to onset of the adjective and baseline corrected before onset of the adjective to avoid a baseline in a region that already differed between conditions.

### Analysis of behavioral data

Behavioral performance was evaluated using mixed-effects logistic regression models with correctness of the response as binary outcome measure (Fitzmaurice et al., 2011). Models were fitted with the glmer command of the lme4 package (Bates et al., 2015) in R (R Core Team, 2016). All models included random intercepts for participant as well as item, considering each unique trial to be a separate item. Significance was evaluated using type-II Wald χ^2^ tests implemented in the car package (Fox and Weisberg, 2011).

It should be noted that the post-target regions of the stimulus sentences differ markedly between sentences with reference-resolving and ambiguous targets. The analysis of behavioral data thus does not directly speak to the difficulty of resolving reference early or late in the sentence, but rather reflects a compound measure of difficulty of the different conditions. The one effect for which conditions were adequately matched concerns the location of the referent. Thus, a significant effect of referent location would indicate that the location of the referent affected the difficulty of the task.

Finally, to test whether the results of the MEG analysis are related to behavioral performance, we computed the correlation between estimated brain activity and the proportion of correct responses. For each subject and each condition, we extracted the mean activation in the spatiotemporal region identified by the significant clusters from the MEG analysis. We then calculated the difference between the response to reference-resolving and ambiguous targets. To test for a correlation across subjects, we correlated this measure with the total proportion of correct responses for each subject. To test for a within-subject correlation, we computed for each subject the correlation between the difference values and proportion correct responses in each condition (three conditions × three referent locations) and submitted the resulting *r* values to a one-sample *t*-test. For determining the proportion of correct responses, we considered only those trials that also entered the MEG analysis.

## Results

### Behavioral performance

Among the participants included in the final analysis, behavioral performance ranged from 71.1% to 98.7% correct answers. A mixed-effects logistic regression model with fixed effects reference (two levels: reference resolved by the target or in the post-target region), modality/target condition (three levels: auditory domain with noun target, visual domain with noun target, or visual domain with adjective target) and location of the referent (three levels) indicated that the effect of condition interacted significantly with reference (χ^2^(2) = 6.54, *p* = 0.038) as well as location (χ^2^(4) = 9.58, *p* = 0.048). To resolve these interactions, we proceeded with a separate analysis for auditory and visual domains.

In auditory domains, there were significant main effects of both reference (χ^2^(1) = 9.78, *p* = 0.002) and location (χ^2^(2) = 13.82. *p* = 0.001). The significant main effect of reference indicated that the percentage of correct responses was higher when reference was resolved early by the noun (*M* = 87.0% correct) than when reference was resolved late, by the prepositional phrase (*M* = 83.8%). This is not unexpected, since questions in the latter condition were slightly more complicated. The main effect of location indicated that responses were more accurate when the referent was the last of the three sounds (*M* = 88.1%) than when it was the second (*M* = 83.5%, *t*(23) = 3.37, *p* = 0.003) or the first (*M* = 84.7%, *t*(23) = 3.02, *p* = 0.006) sound. Behavioral performance thus indicated a recency effect with improved performance on trials in which the referent was the most recently heard sound.

No effects were significant for the visual domains. Overall, responses were more accurate in blocks with visual domains (*M* = 91.1%) than in blocks with auditory domains (*M* = 85.4%, *t*(23) = 6.46, *p* < 0.001).

### Modality-general response

Our primary analysis used a spatiotemporal cluster–based permutation test to find effects associated with reference resolution in the neural response to target words (Fig. 2 and Fig. 3). The test was based on a repeated measures ANOVA with a 2 (reference resolving or ambiguous target) × 3 (condition: auditory domain with noun target, visual domain with noun target, or visual domain with adjective target) × 3 (location of the referent) design. The test was performed in the time window from 200 to 600 ms, and including all parts of the cerebral cortex except the pre- and postrolandic gyri and insula. Results indicated a significant cluster for the main effect of reference in the medial parietal lobe (415–600 ms, *p* = 0.012; Fig. 4). In this region, activation increased whenever the target word resolved reference. Time course plots for the cluster region suggested that this was the case in visual as well as auditory referential domains.

The same test also indicated a main effect of condition at two largely symmetric sites with peaks in the temporal lobes of the two hemispheres (left hemisphere 320–600 ms, *p* < 0.001; right hemisphere 350–600 ms, *p* = 0.009). Pairwise comparison of average activation in the cluster area suggests that after visual domains, the response was higher for nouns than for adjectives, with an even stronger response for nouns after auditory domains. The spatial distribution of the effect resembles previous reports of N400 effects localized with distributed minimum norm estimates of MEG data (Halgren et al., 2002; Lau et al., 2013, 2014). However, that result is difficult to interpret, because a number of variables were not balanced between conditions, leading to differences in lexical frequency and predictability on various levels in addition to possible baseline differences caused by differences between auditory and visual referential domains. In particular, previous research suggests that lexical frequency and predictability interact in influencing N400 amplitudes (Van Petten and Kutas, 1990, 1991; Dambacher et al., 2006). Because of these complications and because this effect of condition does not influence the interpretation of our primary result related to reference resolution, we do not discuss it further here. No other effect in the ANOVA revealed significant clusters.

**Fig. 5.**
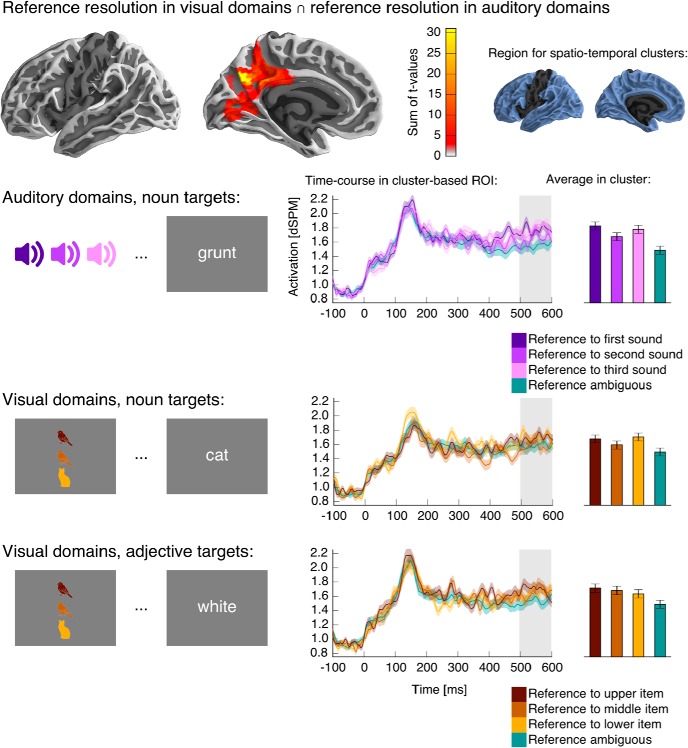
Test for conjunction of reference resolution in auditory and visual referential domains. ***Top***, Anatomical extent of the cluster, showing for each anatomical source the sum of the *t*-values over time, i.e., indicating how much each source contributes to the cluster mass statistic (details analogous to Fig. 4). ***Below***, Activation in the ROI defined by the cluster in the different conditions. ***Left***, Schematic depiction of the referential domains for each condition, and a corresponding example target word. Referents are color-coded for clarity of the results only—referents in the visual referential domains that were presented to subjects were always black and white. ***Middle***, Time course of activation in response to the target word presentation in the ROI defined by the spatial extent of the cluster. The time region covered by the cluster is indicated with gray shading. ***Right***, Bar-plots show the average activation for each condition in the spatiotemporal region covered by the cluster. In both time- and bar-plots, variability is indicated with the within-subject standard error.

Because our study was specifically directed at finding a response to reference resolution that is present for auditory as well as visual domains, we followed up on this initial finding with a test for conjunction of activity related to reference resolution after auditory and visual domains. Results are shown in Fig. 5. The conjunction analysis revealed a single cluster in the medial posterior left hemisphere (500–600 ms, *p* = 0.002) with localization very similar to that of the main effect in the ANOVA. Fig. 5 also displays plots of the activation in the ROI defined by the cluster depending on condition. These plots show that reference resolution was associated with an increase in medial parietal activation across the different conditions.

The ANOVA and the conjunction test results both suggest a shared neural response for reference resolution in auditory and visual domains. A difference in localization and/or timing in the responses should manifest itself in a significant reference × condition interaction, but no such interaction was found. However, this null result could also be because the primary analysis was very conservative. The spatiotemporal cluster–based test, correcting for multiple comparison across a large spatiotemporal region, is maximally sensitive to spatially and temporally extended effects. However, given a largely shared response, an interaction effect reflecting a temporal or spatial difference might be temporally short-lived or spatially constrained. The conservative primary test might thus have missed subtle interaction effects. To test for this possibility, we repeated the ANOVA test, but constrained it to a smaller spatiotemporal region. Spatially, the test was restricted to a medial posterior region in the left hemisphere, only slightly larger than the significant cluster associated with reference resolution. Temporally, the test was restricted to the time window of the ANOVA cluster, i.e., 415–600 ms. No significant reference × condition (or higher-level) interaction was found (all *p* ≥ 0.230). The finding that the conjunction effect starts at 500 ms whereas the ANOVA main effect starts at 415 ms might specifically indicate that the effect differs in onset latency between auditory and visual domains. For an even more liberal test of this hypothesis, we repeated the same ANOVA restricted temporally to the window from 415 to 500 ms, but also this test revealed no significant reference × condition (or higher-level) interaction (all *p* ≥ 0.170). The lack of a significant interaction effect suggests that apparent differences in timing were not reliable. In sum, we found evidence for a shared response to reference resolution in auditory and visual domains in the left medial parietal lobe, and no evidence that this response differs in its spatial or temporal distribution depending on the modality of the referential domain.

MEG activation increase in the medial parietal lobe in association with reference resolution was not significantly correlated with behavioral performance. This was the case for the cluster found in the ANOVA (across subjects, *r*(22) = 0.30, *p* = 0.159; within subjects, mean *r* = –0.09, *t*(23) = –1.25, *p* = 0.223) as well as the cluster from the conjunction analysis (across subjects, *r*(22) = 0.32, *p* = 0.129; within subjects, mean *r* = –0.132, *t*(23) = –1.68, *p* = 0.106).

In the analysis of nouns in adjective-noun phrases (see Fig. 3, bottom) no significant difference was found between reference-resolving and redundant nouns.

### Effects in modality-specific areas

**Fig. 6.**
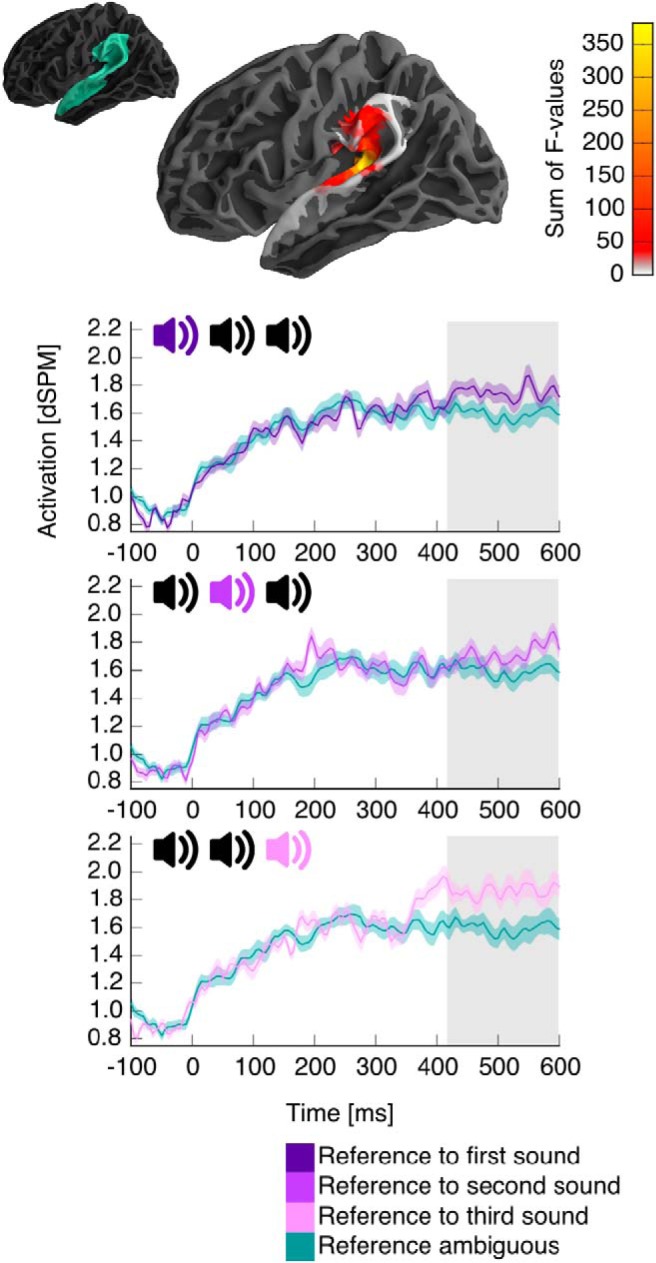
Results in auditory cortex. A significant cluster indicated an activation increase when reference was resolved to auditory objects. The time course of activation in the ROI defined by the cluster is plotted separately according to the sequential position of the referent in the auditory domain. For each plot, the relevant position of the referent is indicated in the schematic of the referential domain. Other details are analogous to previous figures.

We also performed directed tests for activation related to reference resolution in brain regions known to be involved in modality-specific representations. For auditory domains, this included Heschl’s gyrus and the superior temporal and supramarginal gyri of both hemispheres. The 2 (reference) × 3 (location) ANOVA resulted in a cluster with main effect of reference with a maximum in the vicinity of posterior auditory cortex (420–600 ms, *p* < 0.001; Fig. 6). Pairwise comparison suggests that this effect was due to an increase in activation when reference was resolved.

Visual inspection of the time course of activation in Fig. 6 suggests that the time course differed depending on the position of the referent. Although this difference did not result in a significant reference × location interaction at the cluster level, the divergence might simply be too short-lived for the spatiotemporal cluster method, which is maximally sensitive to effects that are extended in space and time. We thus performed a *post hoc* analysis to test for an influence of referent position on the time course of activation. We extracted the average time course of activation for each of the reference-resolving conditions (reference to first, second, and last sound) in the spatial ROI identified by the cluster with main effect of reference. We then performed a temporal cluster-based permutation test with a one-way ANOVA (reference resolution to the first, second, or last sound) in the time window around the onset of main effect of reference resolution, 350–450 ms. The test procedure was analogous to the spatiotemporal cluster test, except that the data was lacking a spatial dimension, and clusters were formed over contiguous time points only. This test revealed a significant effect of referent position (395–420 ms, *p* = 0.037). The average activation in this time window was higher for reference to the last sound compared to the first (*t*(23) = 2.49) as well as the second sound (*t*(23) = 2.42). The first time point at which the activation to reference-resolving nouns differed from the average activation to nonreferential nouns at an uncorrected *p* ≤ 0.05 was 380 ms for reference to the last sound, 490 ms for the second, and 440 ms for the first. This analysis suggests a recency effect, with reference to the most recent sound leading to relatively earlier activation. Although this finding is based on a post hoc test after a more conservative test did not result in a significant interaction effect, and thus requires empirical verification, the finding is consistent with the recency effect found in the behavioral performance data.

The corresponding analysis for activity associated with reference resolution in visual domains in the occipital lobe did not yield any significant clusters.

## Discussion

Our results provide evidence for a brain system recruited during referential language processing that is independent of the modality of the referents and involves the medial parietal lobe. To our knowledge this is the first report of a brain response that is associated with successful reference resolution and not tied to the modality of the referential domain. Crucially, the high temporal resolution of MEG allowed us to attribute this response directly to the reference-resolving words, starting ∼415 ms after word onset. Our results thus go beyond prior hemodynamic studies that implicated the same region in coherent language processing but did not target reference resolution in particular and were unable to attribute the response to a specific stage of sentence comprehension.

Reference resolution is inherently difficult to separate from other cognitive processes associated with managing situation models. At its core, we consider reference resolution to involve identifying an entity in a mental model based on a linguistic description. However, an out-of-the blue reference to an entity that is not immediately present also involves retrieving the relevant entity from memory. For example, when captain Ahab asks a passing ship, “Have ye seen *the White Whale*?” (italics indicate critical expression), he brings to the attention of the addressees an entity that has not been present in the recent discourse or immediate environment. In other situations, reference is made to entities that are not necessarily remembered but are part of the immediate context, as when Ishmael says “Landlord! I’ve changed my mind about that harpooneer. —I shan’t sleep with him. I’ll try *the bench here*.” Although interpreting such a referential expression does not require memory retrieval, it directs attention to the environment for a referent. In the present study, we tried to create an experimental situation that minimizes memory retrieval and scanning of the environment by presenting referential contexts immediately preceding linguistic stimuli that contained referential expressions. The following passage constitutes a textual illustration of such a situation: “The four whales slain that evening had died wide apart; one, far to windward; one, less distant, to leeward; one ahead; one astern. *These last three* were brought alongside ere nightfall; but *the windward one* could not be reached till morning.” Here, memory demands should be minimal because the referents are active immediately before the referential expression, although there might still be additional cognitive processes triggered by accessing the referent—for example, processes related to situating it in the referential domain.

### Modality-general response

Our main result is a response to reference resolution in the medial parietal lobe. We did not find any evidence that this brain response was modulated by the modality of the referents; however, this null result should be interpreted with care. It is possible that the follow-up tests we performed, despite relaxed statistical criteria, were not sensitive enough to detect a subtle effect. For example, it is possible that medial parietal cortex is characterized by a subtle anatomical subdivision with spatially alternating areas that respond to different modalities. Such an effect would be difficult to detect with MEG, which has a source localization accuracy in the order of tens of millimeters (Hauk et al., 2011). Importantly, however, we showed a significant conjunction effect for visual and auditory referents, indicating that even if there might be undetected differences between modalities, the medial parietal lobe, broadly defined, responded to referents in both modalities.

Given the uncertainty inherent in MEG source localization, several regions could be involved in generating the reported reference-sensitive response, including precuneus, posterior cingulate cortex, and retrosplenial cortex. Although anatomical and functional connectivity patterns suggest a more fine-grained division of these regions (Margulies et al., 2009), they are frequently co-activated in fMRI studies (Ranganath and Ritchey, 2012). With this in mind, we will discuss possible connections to other work involving these regions as a group.

A meta-analysis of hemodynamic studies found the left precuneus among the brain regions that were reliably more active for coherent language compared to incoherent language (Ferstl et al., 2008). Most of the included studies compared comprehension of coherent stories (Xu et al., 2005) or sentence pairs (Ferstl and von Cramon, 2002) to unconnected counterparts. Although this is a broad contrast, a crucial component of coherence is repeated reference to the same entities. Our results showing increased activity directly after presentation of a reference-resolving word thus go beyond these previous results and suggest a more specific role for medial parietal cortex in invoking known entities as referents.

Interestingly, we observed the same response not only for complete noun phrases, such as *the bird*, but also for incomplete phrases that provided enough information to resolve reference, such as *the black* in a context with only one black item. This result suggests that this response is associated with reference resolution as a cognitive process that uses information extracted from language, but is not tied by its formal properties, i.e., does not require a complete noun phrase to resolve reference. This result adds neurophysiological evidence for the hypothesis that reference resolution is quick and incremental (Tanenhaus et al., 1995).

An involvement of medial parietal cortex in reference resolution adds a new component to our understanding of medial parietal lobe function. The medial parietal lobe has been consistently implicated in episodic memory. Brain damage involving retrosplenial cortex is associated with episodic memory deficits (Maguire, 2001). In addition, functional imaging studies frequently associated the medial parietal lobe with episodic retrieval. This includes, for example, tasks such as recalling the second word of a learned word pair given the first (Krause, 1999) or recalling some aspect of the context in which an item was previously seen as opposed to merely recognizing that it has been previously presented (Lundstrom, 2003; Lundstrom et al., 2005).

If the medial parietal lobe is associated with retrieving referents, then why were ambiguous expressions, which were compatible with two referents, not associated with more activation than resolving expressions? At least two answers can be conceived of. First, it is possible that our participants did not retrieve referential candidates when faced with temporary referential ambiguity. This would be in contrast to results from visual world studies, in which people listening to referentially ambiguous expressions tend to look at potential referents in a visual display (Tanenhaus et al., 1995). However, this difference could be due to differences between the two paradigms; for example, visual world studies tend to have more complex referential domains, and domains are still available for inspection at the time of comprehension. A second possibility is that multiple referential candidates were in fact retrieved, eliciting medial parietal lobe activation. If this was the case, our results would suggest that resolving reference elicits more activation than retrieving referential candidates.

Like episodic memory tasks, reference resolution entails access to a contextually situated cognitive representation, the referent. Unlike typical episodic memory studies, in our paradigm, presentation of the referential domain and reference resolution were mere seconds apart. Similarly, a temporally separated study phase is not a necessary precondition for medial parietal involvement in processes involving the construction of episodic scenes such as imagining future or fictitious events (Buckner and Carroll, 2007; Schacter and Addis, 2007). In a synthesis of the literature, Ranganath and Ritchey (2012) suggested a functional explanation for these observations. They argued that retrosplenial and parahippocampal cortex, which are functionally connected with precuneus and posterior cingulate cortex (Kahn et al., 2008), are part of a memory system for context memory. In the domain of language processing, this system is thought to be responsible for constructing discourse or situation models (Zwaan, 2015). Because reference resolution involves locating referents in such context representations, our findings provide more specific support for this hypothesis, which has the potential to connect research on language and memory.

This interpretation is also consistent with the results from fMRI investigations of referential processing using text stimuli. One study found that stimuli containing conjoined referents (e.g., *Jeremy and Roger*) were associated with increased activity in the precuneus (Boiteau et al., 2014). A similar parietal response, also extending to more dorsal areas, was observed when a passage repeatedly referred to the same person by name instead of with a pronoun (Almor et al., 2007). The authors argued that, because of the strong expectation that repeated references to the same person should use pronouns, readers temporarily interpreted the repeated name as introducing a new referent, and that parietal regions were involved in handling the additional discourse referent and resolving its coreference with the first name.

Another study localized the response to sentences containing referentially ambiguous pronouns, as in *Ronald told Frank that he …*, and found a strong peak in the medial parietal lobe (Nieuwland et al., 2007). Superficially, this is the opposite of our finding, which was more medial parietal activity for unambiguous reference. However, our stimuli contained only temporary ambiguities that were resolved after at most three additional words (*the grunt at the beginning*), whereas the fMRI study involved permanent ambiguities that were never resolved. The low temporal resolution of fMRI cannot distinguish between a direct response to the ambiguous pronouns and a later, possibly extended response to ambiguous sentences. Indeed, the BOLD signal was modeled as an extended response from the critical pronoun up to the onset of the sentence final word (p. 996). Rather than contradicting our results, this finding thus suggests that medial parietal lobe activation not only is involved when a referent is found, but also can be recruited in situations that require dealing with openly ambiguous referential expressions, which might reflect an extended search through a situation model for possible referents. Finally, a study that focused on explicit pronoun coreference judgments implicated in particular the lateral frontal lobes for processing of ambiguities, which could thus be related to higher-level decision-making demands (McMillan et al., 2012). Together, these results suggest a more complex picture of the processing of referential ambiguities with functionally separable contributions from different brain regions.

Our design resembles short-term memory tasks, in which a recognition task follows presentation of study items after only seconds. This analogy suggests the alternative possibility that the medial parietal lobe is involved merely in recalling the spatiotemporal properties of the referents. Although our design by itself cannot exclude this possibility, it is rendered less plausible when considering other research: short-term memory tasks for visual features tend to engage lateral rather than medial parietal regions (Todd and Marois, 2004; Kawasaki et al., 2008; Bettencourt and Xu, 2015), nor do auditory short-term memory tasks tend to engage the medial parietal lobe (Kumar et al., 2016). On the other hand, medial parietal regions are recruited by tasks that involve judgments of complex spatial or temporal relations (Galati et al., 2010; Kwok and Macaluso, 2015), which is more consistent with an involvement in relational models as argued above. Furthermore, an explanation based solely on short-term memory access might have difficulty accounting for results from the hemodynamic studies discussed above, which implicated the medial parietal lobe in referential language processing while using linguistic stimuli.

More generally, it could be argued that the medial parietal lobe is involved in encoding perceptual as opposed to semantic relations. This explanation could account for fMRI studies of language processing, assuming that participants in those studies maintained perceptual models during language comprehension. This assumption is reasonable given evidence that even purely linguistic stimuli seem to be encoded involving modality-specific systems (see sources cited in the Introduction). At least, the evidence presented here suggests that representations in the medial parietal lobe are not purely visual, but multisensory. This issue also highlights a larger question for future research concerning the extent to which sensory representations are essential to discourse models, or whether they are epiphenomenal.

A further possibility is that the medial parietal lobe response reflects a priming effect of the ambiguous targets, because ambiguous targets were applicable to two entities in the referential domain, whereas reference-resolving words were applicable to only one. We consider this explanation unlikely, because semantic priming effects tend to start earlier, even when sounds prime words (Vanpetten and Rheinfelder, 1995), and localize to temporal and sometimes frontal regions (Lau et al., 2008; Lau et al., 2013, 2014). Both characteristics apply to the N400-like responses we observed as main effects between target conditions, suggesting that we did have the power to detect such priming effects, had they been present.

In sum, our results, together with the literature on the medial parietal lobe, suggest that this region is involved in representing entities with contextual associations, and thus in maintaining situation models for language comprehension.

### Activity in modality-specific brain systems

Our results suggest that resolving reference to a previously heard sound is associated with an increase in activation in the vicinity of auditory cortex. This response occurred in the absence of concurrent auditory stimulation, suggesting that it is associated with retrieval of sensory properties of the referent. More detailed analysis suggested that reference to the most recent sound was associated with faster activation, consistent with theories suggesting that the most recent in a sequence of items stored in short-term memory is more accessible (McElree and Dosher, 1989).

The localization of this effect in the vicinity of auditory cortex is compatible with auditory imagery, which is associated with activation posterior to Heschl’s gyrus bilaterally (McNorgan, 2012), although the same regions might also encode more abstract information (Linke and Cusack, 2015). In our study, the response was left-lateralized, paralleling neuroimaging (McNorgan, 2012) as well as behavioral (Prete et al., 2016) evidence for a tendency toward functional left-lateralization of imagery.

Given that this response likely reflects activation of modality-specific representations, the direction of the effect is interesting. Ambiguous nouns were compatible with two auditory objects; activating two tokens as part of the referential search could lead to more activity than activating a single token. Instead, we found that auditory cortex becomes more active once a unique referent is found. This result suggests that auditory cortex becomes more involved not during the search for a referent but when the referent is found, consistent with the need to retrieve a more detailed representation of the referent to answer the question.

The parallel test for activity associated with reference resolution to visual referents did not reveal any significant clusters. Although one possibility is that visual referential domains were processed differently from the auditory domains, there are other possible explanations for this null result. Processing the visually presented words could have interfered with, or overshadowed, a response associated with the referential domain. Reference resolution in auditory domains was not associated with such a competing process, as words were presented visually. Furthermore, previous results suggest that reference resolution in visual domains is associated with a brain response that depends on the spatial position of the referent along the horizontal axis (Brodbeck et al., 2015), consistent with results indicating that visual short-term memory access is associated with a brain response that depends on the horizontal position of the item that is accessed (Hopf et al., 2000; Hopf et al., 2004). However, in the present study, referents were arranged vertically, and to our knowledge, location-dependent activity in the vertical dimension is not an established phenomenon.

Although the behavioral results paralleled the recency-effect observed in the auditory domains, with better performance when the referent was the most recently presented sound, the location of the referent did not influence behavioral performance in visual domains. This could reflect the fact that in visual domains all referents were presented concurrently, contrasting with the serial presentation of the auditory referents.

### Models of language comprehension

The response in auditory cortex started at practically the same time as the medial parietal, modality-general response; if considering reference to the most recent sound, it preceded it. This observation constrains the functional interpretation of the two responses. Specifically, the medial parietal response does not seem to be the earliest response reflecting reference resolution. On the other hand, we cannot exclude the possibility that reference resolution involves modality-specific systems, whereas the medial parietal response reflects a subsequent process such as integrating the referent in a situation model.

In the context of models of visual word perception (e.g. Pylkkänen and Marantz, 2003; Grainger and Holcomb, 2009) the onset of ∼400 ms puts our effects in a postlexical time window. This is in agreement with a sequential model in which reference resolution follows lexical processing. Consistent with this, we observed an N400-like response with an onset of ∼320 ms. Given that the N400 is thought to reflect access to lexical information (Lau et al., 2014), activation of lexical information is suggested to precede reference resolution.

By describing a neural response to reference resolution, our results add a critical component to the sequence of computational steps in language comprehension that can be tracked with electrophysiology. Our results thus offer not only novel insights into the neural basis of reference resolution, but also new tools to study language processing.

## Conclusions

Our results provide evidence against a model of referential language processing in which semantic language representations interact with representations of the referential domain exclusively in modality-specific brain systems. Instead, our findings suggest that a brain system including a medial parietal region supports referential language processing with an increase in activity when the referent of an expression can be resolved. This finding provides a crucial bridge between language processing and the memory literature, which attributes context representations based on situation models to medial parietal brain regions (Ranganath and Ritchey, 2012). In addition, our results provide a possible explanation for the consistent observation of medial parietal activity during tasks involving coherent language (Nieuwland et al., 2007; Ferstl et al., 2008; Boiteau et al., 2014). Although our finding thus provides a considerable advance in region-function mapping, reference resolution is not a monolithic process, and it will be a task for future research to clarify the precise computational steps that engage medial parietal and modality-specific regions.

## Synthesis

Reviewing Editor: Lila Davachi, New York University Decisions are customarily a result of the Reviewing Editor and the peer reviewers coming together and discussing their recommendations until a consensus is reached. When revisions are invited, a fact-based synthesis statement explaining their decision and outlining what is needed to prepare a revision will be listed below. The following reviewer(s) agreed to reveal their identity: Fernanda Ferreira, Amit Almor.

There is agreement that the results are interesting. However, both reviewers also agreed that there were limitations that could be addressed in three ways: (1) further statistical analyses, (2) presentation of additional behavioral data from the study and (3) tempered claims about the findings. I will address each of these below using specific comments from the reviews.

Statistical Analyses:

1) In some places null effects are interpreted as indicating the absence of an effect rather than merely a failure to find one. This is critical for one of the main claims of the paper which is the amodal activation associated with reference resolution. This should be approached with greater care and explicitly discussed.

2) "The behavioral data analyses showed some critical differences between the modalities. Firstly, I would have liked to see an item analysis of these results as is customary in analyses of behavioral psycholinguistic data. Alternatively, the authors could use mixed models which have become the de facto standard for such analyses. Second, given the marked behavioral differences between the conditions I think that it is important to consider the MEG data with respect to the behavioral performance. Otherwise it is difficult to tell whether differences, or lack of differences between the modalities in the main MEG data are driven by overall difficulty and accuracy."

3) "s in any analysis that involves brain localization of multiple subjects, the results can be radically affected by the registration and normalization methods used. It will be helpful to have some justification and references to research that validates the methods and atlas used for the source localization and labeling."

Temper claims:

1) Both reviewers were concerned that there were other processes, besides reference resolution, per se, that could be responsible for the MEG results. They both point to working memory and updating processes that might be consistent with the medial parietal localizations reported here. One reviewer states that 'given that the task is essentially a memory task where participants have to respond to a prompt based on a memorized representation of the stimuli, the observed activations could reflect the working memory processes that follow the resolution of the reference (for example, the updating of working memory that can occur in this task immediately after the reference is resolved, or the beginning of forming a response to the question). Indeed, in addition to their involvement in processing coherent discourse and reference processing, medial parietal regions are known to be involved in working memory, aspects of which are inherently amodal. Furthermore, the various recency effects observed in the auditory condition (which was the only one in which stimuli were presented sequentially) further highlight the role of working memory in this task and its potential influence on the results. Similar working memory processes likely occurred in the visual condition although they were not likely to be reflected in order effects since in these conditions the stimuli, as far as I understand, were presented simultaneously. I think that whether or not the authors are able to argue against such alternative interpretations (memory updating and/or action planning), they should be considered and discussed in the paper. " The second reviewer states 'the medial parietal lobe is known be involved in spatiotemporal processing and retrieval of explicit episodic source information (e.g., Lundstrom et al., 2003; 2005). Since the experimental task always involved finding the location of the referent among two other competing referential candidates along either temporal or spatial dimensions, it might not be surprising that this area becomes activated for both modalities. The cognitive process underlying the task might have been not so much resolving anaphoric dependencies as they occur during normal language processing (i.e., maintaining the semantic information of preceding referential candidates and re-activating and binding them upon encountering the anaphor), but simply memorizing and recalling the spatiotemporal properties of the potential referents. The fact that there are no fillers in the experiment further strengthens the possibility that the task was probably performed strategically, most likely by tagging each potential referent with its respective location. " This reviewer goes on to state 'The authors found that unambiguous (noun resolving) anaphors were more difficult to process than ambiguous (noun non-resolving) anaphors. This goes against many ERP and behavioral findings that ambiguous anaphor resolution is more difficult than unambiguous anaphor resolution (e.g., Nieuwland and van Berkum, 2006; Rigalleau & Caplan, 2000). This effect, too, seems to arise from the possibility that participants are perhaps merely tagging each object with a location. When the anaphor is unambiguous, the location information associated with the target object is retrieved, producing the observed difficulty upon encountering the anaphor. However, when the anaphor is ambiguous the location cannot and is not retrieved. Thus, participants perhaps simply wait until disambiguating information is available before deciding which object it is that they need to retrieve the location for. Note how this type of task differs from normal anaphora processing which involves retrieving the intrinsic semantic content of referential candidates rather than tagged, non-intrinsic information such as location. " It is important that you discuss potential alternative interpretations for your results.

2) Additional possibilities were offered as well that should be addressed: "Another possible reason why ambiguous anaphors were processed more easily than unambiguous anaphors could be that ambiguous anaphors received activation from two matching objects but unambiguous anaphors received activation from only one associated object. This is because the potential referent (i.e., the sound of grump or the picture of the bird) was presented twice (auditorily or visually) in the ambiguous condition but only once in the unambiguous condition. Thus, facilitated anaphor processing for ambiguous anaphors could be due to a priming effect. The authors refute a lexical facilitation account by arguing that the effect emerges too late for it be lexical access (Pylkkänen & Marantz, 2003). However, the cited studies (and most other studies) investigating the time course of lexical access are based on linguistic stimuli. Lexical facilitation from sounds/pictures to words could occur later because the lexeme information for words needs to be activated before priming could occur.

3) "The theoretical hypotheses in the introduction are a bit misrepresented and from the real value of this paper which is in providing information about the neural bases of reference resolution. In my understanding, there is really no one (including the cited Pulvermuller, 2013) who argues that there is not *some* amodal aspect to language processing (counter to the claim in line 79 for example), or that there are not parietal regions that are amodal, so this is somewhat of a straw man hypothesis. The argument is really about whether the modality specific representations that have been reported by multiple studies are essential for language representation and, in the current context, for referent representation, or are they epiphenomenal and not critical. For that reason, these particular theoretical hypotheses are not quite relevant to this study, which does not provide a critical test of any of them. I think that the introduction could be strengthened if these ideas are only mentioned in so far as they serve the goal of explaining what reference resolution is and how it might differ as function of the modality of referent. "

(6) "The auditory vs visual comparison is confounded by the different word frequencies of the stimuli (as is acknowledged by the authors), differences in sequential vs simultaneous presentation of the stimuli (auditory stimuli can be presented only sequentially but the visual stimuli could have also been presented sequentially to match the presentation of the auditory items), and the fact that the names of the auditory items but not the visual items had two shared onsets (/b/* and /c/*). A really picky point - the choice of the /b/ sound is not ideal if any of the participants were Arabic speakers as Arabic does not phonemically distinguish between /b/ and /p/. The clear differences in difficulty between the visual and auditory tasks could be one reason for why the auditory items led to differential activation in auditory cortex but the visual items did not lead to differential activation in visual cortex.

(7) There are a large number of post hoc tests that are conducted on a portion of the data that a previous analysis revealed has a significant effect (for example an analysis of an interaction effect in a region that previously showed a main effect, or an analysis of only a time window that was found significant in an earlier analysis). The authors should be more careful about interpreting the results of these analyses. "

More data:

1. One of the reviewers was also concerned about yet another alternative possibility: "ambiguous anaphors may have been resolved but were resolved late. In fact, there is evidence for delayed reference resolution in self-paced and visual world paradigms (e.g., Stewart et al., 2007; Karimi & Ferreira, 2015). This delaying strategy for ambiguous anaphors seems even more likely considering that ambiguous anaphors were always disambiguated at the end of the sentence. As such, participants might have learned over the course of the experiment to wait for disambiguating information when the anaphor is ambiguous. I think the authors need to examine their data at later windows too to see if there are effects there. Related to this, I think the authors should provide reaction time data for their behavioral results. If unambiguous anaphors were harder to process in their design, it should also take longer for participants to respond to the questions. I am assuming they have the reaction time data as well as accuracy." Please provide additional behavioral data to examine if this is a possibility.

2. It is also requested that you report the results from the reverse analysis (ambiguous minus unambiguous) as the reverse comparison could provide valuable information as to which brain areas become activated when the anaphor is not resolved. It was noted that "Nieuwland et al. (2007), for example, found a double dissociation: Referential ambiguity resulted in reduction of activation in LIFG and a boost of activation in medial prefrontal and parietal regions."

3. It was additionally pointed out that there are main effects of anaphor ambiguity as well as location in the auditory condition but no such effects are found in the visual condition. This could potentially argue against a modality-independent referential processing. The authors need to discuss why there are behavioral differences between modalities and how this could be justified under a modality-independent account. It could be the case that retrieving referential candidates from a temporal-dimension is relatively more similar to normal referential processing than retrieving from a spatial dimension, which in turn could be the reason why modality-specific effects are found for auditory but not for visual stimuli. Again, the absence of the two main effects in the visual condition (that are present in the auditory condition) raises even more concerns about whether the cognitive processing in this domain was in fact similar to referential processing as it happens during normal comprehension.

Additional comments

1. The labels for the conditions should be changed to make them clearer. In particular, I recommend changing the label representing modality from "condition" to "modality". I also recommend changing "noun-(non)-resolving" to "(un)ambiguous".

2. The authors should provide Brodman area information on the regions of interest (in addition to labels) to maximize transparency.

3. (4) "Lines 84-99: It will be helpful to reference and review here the fMRI studies that did examine reference processing, especially the ones that revealed parietal activation. Some of these studies are mentioned sporadically later in the paper, but it will be helpful for the reader to know at the onset what is already known about reference processing in the parietal lobe (the studies by Almor, Boiteau, Nieuwland, and also by McMillan)

4. "Participants (113): Given that the study was run in the Abu Dhabi campus, it will be useful to include more information about the linguistic background of the participants. Where some of them bilingual? Where they immersed in an Arabic speaking environment prior to the study, etc? This is especially important because Arabic is written right to left and has a different adjective-noun order then English. Both may have an effect on at least some of the conditions in this experiment."

## References

[B1] Adachi, Y., Shimogawara M., Higuchi M., Haruta Y., Ochiai M. (2001). Reduction of non-periodic environmental magnetic noise in MEG measurement by continuously adjusted least squares method. IEEE Transact Appl Superconduct 11:669–672. 10.1109/77.919433

[B2] Almor A., Smith D. V., Bonilha L., Fridriksson J., Rorden C. (2007). What is in a name? Spatial brain circuits are used to track discourse references. Neuroreport 18:1215–1219. 10.1097/WNR.0b013e32810f2e11 17632270

[B3] Altmann G. T. M. (2004). Language-mediated eye movements in the absence of a visual world: the “blank screen paradigm.” Cognition 93:B79–B87. 10.1016/j.cognition.2004.02.005 15147941

[B4] Altmann G. T. M., Kamide Y. (2009). Discourse-mediation of the mapping between language and the visual world: eye movements and mental representation. Cognition 111:55–71. 19193366 10.1016/j.cognition.2008.12.005 PMC2669403

[B5] Barsalou L. W. (1999). Perceptions of perceptual symbols. Behav Brain Sci 22:637–660. 10.1017/S0140525X99532147 11301525

[B6] Bates D., Mächler M., Bolker B., Walker S. (2015). Fitting linear mixed-effects models using lme4. J Stat Softw 67:1–48. 10.18637/jss.v067.i01

[B7] Bemis D. K., Pylkkänen L. (2011). Simple composition: a magnetoencephalography investigation into the comprehension of minimal linguistic phrases. J Neurosci 31:2801–2814. 10.1523/JNEUROSCI.5003-10.2011 21414902 PMC6623787

[B8] Bettencourt K. C., Xu Y. (2015). Decoding the content of visual short-term memory under distraction in occipital and parietal areas. Nature Neurosci 19:150–157. 10.1038/nn.4174 26595654 PMC4696876

[B9] Bizley J. K., Cohen Y. E. (2013). The what, where and how of auditory-object perception. Nat Rev Neurosci 14:693–707. 10.1038/nrn3565 24052177 PMC4082027

[B10] Boiteau T. W., Bowers E., Nair V. A., Almor A. (2014). The neural representation of plural discourse entities. Brain Lang 137:130–141. 10.1016/j.bandl.2014.08.003 25218099

[B11] Bransford J. D., Johnson M. K. (1972). Contextual prerequisites for understanding: some investigations of comprehension and recall. J Verbal Learn Verbal Behav 11:717–726. 10.1016/S0022-5371(72)80006-9

[B12] Brodbeck C., Gwilliams L., Pylkkänen L. (2015). EEG can track the time course of successful reference resolution in small visual worlds. Front Psychol 6:1787. 10.3389/fpsyg.2015.01787 26635689 PMC4653275

[B13] Brysbaert M., New B. (2009). Moving beyond Kucera and Francis: a critical evaluation of current word frequency norms and the introduction of a new and improved word frequency measure for American English. Behav Res Methods 41:977–90. 10.3758/BRM.41.4.977 19897807

[B14] Buckner R. L., Carroll D. C. (2007). Self-projection and the brain. Trends Cogn Sci 11:49–57. 10.1016/j.tics.2006.11.004 17188554

[B15] Dale A. M., Liu A. K., Fischl B. R., Buckner R. L., Belliveau J. W., Lewine J. D., Halgren E. (2000). Dynamic statistical parametric mapping: combining fMRI and MEG for high-resolution imaging of cortical activity. Neuron 26:55–67. 10.1016/S0896-6273(00)81138-1 10798392

[B16] Dambacher M., Kliegl R., Hofmann M., Jacobs A. M. (2006). Frequency and predictability effects on event-related potentials during reading. Brain Res 1084:89–103. 10.1016/j.brainres.2006.02.010 16545344

[B17] Desikan R. S., Ségonne F., Fischl B., Quinn B. T., Dickerson B. C., Blacker D., … Killiany R. J. (2006). An automated labeling system for subdividing the human cerebral cortex on MRI scans into gyral based regions of interest. NeuroImage 31:968–980. 10.1016/j.neuroimage.2006.01.021 16530430

[B18] Ding N., Simon J. Z. (2012). Emergence of neural encoding of auditory objects while listening to competing speakers. Proc Natl Acad Sci U S A 109:11854–11859. 10.1073/pnas.1205381109 22753470 PMC3406818

[B19] Eberhard K. M., Spivey-Knowlton M. J., Sedivy J. C., Tanenhaus M. K. (1995). Eye movements as a window into real-time spoken language comprehension in natural contexts. J Psycholinguist Res 24:409–436. 10.1007/BF02143160> 8531168

[B20] Ferstl E. C., Neumann J., Bogler C., von Cramon D. Y. (2008). The extended language network: a meta-analysis of neuroimaging studies on text comprehension. Hum Brain Mapping 29:581–593. 10.1002/hbm.20422 > 17557297 PMC2878642

[B21] Ferstl E. C., von Cramon D. Y. (2002). What does the frontomedian cortex contribute to language processing: coherence or theory of mind? NeuroImage 17:1599–1612. 10.1006/nimg.2002.1247 12414298

[B22] Fitzmaurice G. M., Laird N. M., Ware J. H. (2011). Appl Longitudinal Analysis (2nd ed). Hoboken, N.J: Wiley.

[B23] Fox J., Weisberg S. (2011). An R Companion to Applied Regression (Second). Thousand Oaks CA: Sage Retrieved from http://socserv.socsci.mcmaster.ca/jfox/Books/Companion

[B24] Galati G., Pelle G., Berthoz A., Committeri G. (2010). Multiple reference frames used by the human brain for spatial perception and memory. Exp Brain Res 206:109–120. 10.1007/s00221-010-2168-8 20186405

[B25] Giordano B. L., McAdams S., Zatorre R. J., Kriegeskorte N., Belin P. (2013). Abstract encoding of auditory objects in cortical activity patterns. Cereb Cortex 23:2025–2037. 10.1093/cercor/bhs162 22802575

[B26] Graesser A. C., Millis K. K., Zwaan R. A. (1997). Discourse comprehension. Ann Rev Psychol 48:163–189. 10.1146/annurev.psych.48.1.163 15012477

[B27] Grainger J., Holcomb P. J. (2009). Watching the word go by: on the time-course of component processes in visual word recognition. Lang Linguist Compass 3:128–156. 10.1111/j.1749-818X.2008.00121.x 19750025 PMC2740997

[B28] Gramfort A., Luessi M., Larson E., Engemann D. A., Strohmeier D., Brodbeck C., … Hämäläinen M. S. (2013). MEG and EEG data analysis with MNE-Python. Front Neurosci 7:267. 10.3389/fnins.2013.00267 24431986 PMC3872725

[B29] Gramfort A., Luessi M., Larson E., Engemann D. A., Strohmeier D., Brodbeck C., Hämäläinen M. S. (2014). MNE software for processing MEG and EEG data. NeuroImage 86:446–460. 10.1016/j.neuroimage.2013.10.027 24161808 PMC3930851

[B30] Halgren E., Dhond R. P., Christensen N., Van Petten C., Marinkovic K., Lewine J. D., Dale A. M. (2002). N400-like magnetoencephalography responses modulated by semantic context, word frequency, and lexical class in sentences. NeuroImage 17:1101–1116. 10.1006/nimg.2002.1268 12414253

[B31] Hauk O., Coutout C., Holden A., Chen Y. (2012). The time-course of single-word reading: evidence from fast behavioral and brain responses. NeuroImage 60:1462–1477. 10.1016/j.neuroimage.2012.01.061 > 22281671 PMC3382728

[B32] Hauk O., Davis M. H., Kherif F., Pulvermüller F. (2008). Imagery or meaning? Evidence for a semantic origin of category-specific brain activity in metabolic imaging. Eur J Neurosci 27:1856–1866. 10.1111/j.1460-9568.2008.06143.x 18380676 PMC2327213

[B33] Hauk O., Wakeman D. G., Henson R. N. (2011). Comparison of noise-normalized minimum norm estimates for MEG analysis using multiple resolution metrics. NeuroImage 54:1966–1974. 10.1016/j.neuroimage.2010.09.053 20884360 PMC3018574

[B34] Holmes A. P., Blair R. C., Watson G., Ford I. (1996). Nonparametric analysis of statistic images from functional mapping experiments. J Cereb Blood Flow Metab 16:7–22. 10.1097/00004647-199601000-00002 8530558

[B35] Hopf J. M., Boelmans K., Schoenfeld M. A., Luck S. J., Heinze H. J. (2004). Attention to features precedes attention to locations in visual search: evidence from electromagnetic brain responses in humans. J Neurosci 24:1822–1832. 10.1523/JNEUROSCI.3564-03.2004 14985422 PMC6730400

[B36] Hopf J. M., Luck S. J., Girelli M., Hagner T., Mangun G. R., Scheich H., Heinze H. J. (2000). Neural sources of focused attention in visual search. Cereb Cortex 10:1233–1241. 10.1093/cercor/10.12.1233 11073872

[B37] Kahn I., Andrews-Hanna J. R., Vincent J. L., Snyder A. Z., Buckner R. L. (2008). Distinct Cortical Anatomy Linked to Subregions of the Medial Temporal Lobe Revealed by Intrinsic Functional Connectivity. J Neurophysiol 100:129–139. 10.1152/jn.00077.2008 18385483 PMC2493488

[B38] Kawasaki M., Watanabe M., Okuda J., Sakagami M., Aihara K. (2008). Human posterior parietal cortex maintains color, shape and motion in visual short-term memory. Brain Res 1213:91–97. 10.1016/j.brainres.2008.03.037 18455152

[B39] Krause B. J. (1999). Episodic retrieval activates the precuneus irrespective of the imagery content of word pair associates: a PET study. Brain 122:255–263. 10.1093/brain/122.2.255 10071054

[B40] Kumar S., Joseph S., Gander P. E., Barascud N., Halpern A. R., Griffiths T. D. (2016). A brain system for auditory working memory. J Neurosci 36:4492–4505. 10.1523/JNEUROSCI.4341-14.2016 27098693 PMC4837683

[B41] Kwok S. C., Macaluso E. (2015). Immediate memory for “when, where and what”: short-delay retrieval using dynamic naturalistic material: neural correlates of immediate episodic retrieval. Hum Brain Mapping 36:2495–2513. 10.1002/hbm.22787 PMC5006857 25773646

[B42] Lau E. F., Gramfort A., Hamalainen M. S., Kuperberg G. R. (2013). Automatic semantic facilitation in anterior temporal cortex revealed through multimodal neuroimaging. J Neurosci 33:17174–17181. 10.1523/JNEUROSCI.1018-13.2013 24155321 PMC3807034

[B43] Lau E. F., Phillips C., Poeppel D. (2008). A cortical network for semantics: (de)constructing the N400. Nat Rev Neurosci 9:920–933. 10.1038/nrn2532 19020511

[B44] Lau E. F., Weber K. P., Gramfort, A., Hamalainen M. S., Kuperberg G. R. (2014). Spatiotemporal signatures of lexical-semantic prediction. Cereb Cortex 26:1377–1387. 10.1093/cercor/bhu219 25316341 PMC4785937

[B45] Linke A. C., Cusack R. (2015). Flexible information coding in human auditory cortex during perception, imagery, and STM of complex sounds. J Cogn Neurosci 27:1322–1333. 10.1162/jocn_a_00780 25603030

[B46] Loftus G. R., Masson M. E. J. (1994). Using confidence intervals in within-subject designs. Psychonom Bull Rev 1:476–490. 10.3758/BF03210951 24203555

[B47] Lundstrom B. N. (2003). Isolating the retrieval of imagined pictures during episodic memory: activation of the left precuneus and left prefrontal cortex. NeuroImage 20:1934–1943. 10.1016/j.neuroimage.2003.07.017 14683699

[B48] Lundstrom B. N., Ingvar M., Petersson K. M. (2005). The role of precuneus and left inferior frontal cortex during source memory episodic retrieval. NeuroImage 27:824–834. 10.1016/j.neuroimage.2005.05.008 15982902

[B49] Maguire E. A. (2001). The retrosplenial contribution to human navigation: a review of lesion and neuroimaging findings. Scand J Psychol 42:225–238. 10.1111/1467-9450.00233 11501737

[B50] Margulies D. S., Vincent J. L., Kelly C., Lohmann G., Uddin L. Q., Biswal B. B., … Petrides M. (2009). Precuneus shares intrinsic functional architecture in humans and monkeys. Proc Natl Acad Sci U S A 106:20069–20074. 10.1073/pnas.0905314106 19903877 PMC2775700

[B51] Maris E., Oostenveld R. (2007). Nonparametric statistical testing of EEG- and MEG-data. J Neurosci Methods 164:177–190. 10.1016/j.jneumeth.2007.03.024 17517438

[B52] McElree B., Dosher B. A. (1989). Serial position and set size in short-term memory: the time course of recognition. J Exp Psychol Gen 118:346–373. 10.1037//0096-3445.118.4.346

[B53] McMillan C. T., Clark R., Gunawardena D., Ryant N., Grossman M. (2012). fMRI evidence for strategic decision-making during resolution of pronoun reference. Neuropsychologia 50:674–687. 10.1016/j.neuropsychologia.2012.01.004 22245014 PMC3309154

[B54] McNorgan C. (2012). A meta-analytic review of multisensory imagery identifies the neural correlates of modality-specific and modality-general imagery. Front Hum Neurosci 6:285 10.3389/fnhum.2012.00285 23087637 PMC3474291

[B55] Nichols T. E., Brett M., Andersson J., Wager T. D., Poline J. B. (2005). Valid conjunction inference with the minimum statistic. NeuroImage 25:653–60. 10.1016/j.neuroimage.2004.12.005 15808966

[B56] Nieuwland M. S., Petersson K. M., Van Berkum J. J. A. (2007). On sense and reference: examining the functional neuroanatomy of referential processing. NeuroImage 37:993–1004. 10.1016/j.neuroimage.2007.05.048 17611124

[B57] Patterson K., Nestor P. J., Rogers T. T. (2007). Where do you know what you know? The representation of semantic knowledge in the human brain. Nat Rev Neurosci 8:976–87. 10.1038/nrn2277 18026167

[B58] Prete G., Marzoli D., Brancucci A., Tommasi L. (2016). Hearing it right: evidence of hemispheric lateralization in auditory imagery. Hear Res 332:80–86. 10.1016/j.heares.2015.12.011 26706706

[B59] Pulvermuller F. (2013). How neurons make meaning: brain mechanisms for embodied and abstract-symbolic semantics. Trends Cogn Sci 17:458–70. 10.1016/j.tics.2013.06.004 23932069

[B60] Pylkkänen L., Marantz A. (2003). Tracking the time course of word recognition with MEG. Trends Cogn Sci 7:187–189. 10.1016/S1364-6613(03)00092-5 12757816

[B61] R Core Team. (2016). R: A language and environment for statistical computing. Vienna, Austria: R Foundation for Statistical Computing Retrieved from https://www.R-project.org/

[B62] Ranganath C., Ritchey M. (2012). Two cortical systems for memory-guided behaviour. Nat Rev Neurosci 13:713–726. 10.1038/nrn3338 22992647

[B63] Schacter D. L., Addis D. R. (2007). The cognitive neuroscience of constructive memory: remembering the past and imagining the future. Philos Trans R Soc B Biol Sci 362:773–786. 10.1098/rstb.2007.2087 PMC2429996 17395575

[B64] Sedivy J. C. K., Tanenhaus M., Chambers C. G., Carlson G. N. (1999). Achieving incremental semantic interpretation through contextual representation. Cognition 71:109–147. 10.1016/S0010-0277(99)00025-6 10444906

[B65] Spivey M. J., Geng J. J. (2001). Oculomotor mechanisms activated by imagery and memory: eye movements to absent objects. Psychol Res 65:235–241. 10.1007/s004260100059 11789427

[B66] Spivey M. J., Tanenhaus M. K., Eberhard K. M., Sedivy J. C. (2002). Eye movements and spoken language comprehension: effects of visual context on syntactic ambiguity resolution. Cogn Psychol 45:447–481. 10.1016/S0010-0285(02)00503-0 12480476

[B67] Tanenhaus M. K., Spivey-Knowlton M. J., Eberhard K. M., Sedivy J. C. (1995). Integration of visual and linguistic information in spoken language comprehension. Science 268:1632–1634. 10.1126/science.7777863 7777863

[B68] Todd J. J., Marois R. (2004). Capacity limit of visual short-term memory in human posterior parietal cortex. Nature 428:751–754. 10.1038/nature02466 15085133

[B69] Van Essen D. C. (2005). A population-average, landmark- and surface-based (PALS) atlas of human cerebral cortex. NeuroImage 28:635–662. 10.1016/j.neuroimage.2005.06.058 16172003

[B70] Van Petten C., Kutas M. (1990). Interactions between sentence context and word frequency in event-related brain potentials. Mem Cogn 18:380–393. 10.3758/BF03197127 2381317

[B71] Van Petten C., Kutas M. (1991). Influences of semantic and syntactic context on open- and closed-class words. Mem Cogn 19:95–112. 10.3758/BF03198500 2017035

[B72] Vanpetten C., Rheinfelder H. (1995). Conceptual relationships between spoken words and environmental sounds: event-related brain potential measures. Neuropsychologia 33:485–508. 10.1016/0028-3932(94)00133-A 7617157

[B73] Xu J., Kemeny S., Park G., Frattali C., Braun A. (2005). Language in context: emergent features of word, sentence, and narrative comprehension. NeuroImage 25:1002–1015. 10.1016/j.neuroimage.2004.12.013 15809000

[B74] Zwaan R. A. (2015). Situation models, mental simulations, and abstract concepts in discourse comprehension. Psychon Bull Rev 10.3758/s13423-015-0864-x PMC4974264 26088667

